# Mamba-YOLO-SRC: An Automatic Deep Learning Framework for Respiratory Behavior Detection in the Chinese Giant Salamander

**DOI:** 10.3390/ani16121923

**Published:** 2026-06-22

**Authors:** Dingwei Mao, Yan Zhou, Chenyang Shi, Xinyuan Zhang, Guanglin Chen, Yuanqiong Chen, Qinghua Luo

**Affiliations:** 1 School of Computer Science and Engineering, Jishou University, Jishou 416000, China; 2 College of Life Sciences, Jishou University, Jishou 416000, China; 3 Hunan Engineering Technology Research Center for Amphibian and Reptile Resource Protection and Product Processing, College of Biological and Chemical Engineering, Changsha University, Changsha 410017, China

**Keywords:** respiratory behavior, *Andrias davidianus*, YOLO, hybrid detection model, deep learning

## Abstract

The Chinese giant salamander (*Andrias davidianus*), a species of high ecological and conservation value, shows abnormal respiratory behaviors as early signs of health decline. Accurate assessment of its pulmonary respiration is crucial for improving captive breeding and post-breeding parental care—key strategies for its survival and population recovery. However, its nocturnal and cave-dwelling nature makes traditional observation extremely difficult. Manual monitoring suffers from poor visibility at night, while conventional detection methods often miss subtle respiratory movements, limiting behavioral and health research. To address these challenges, this study presents the first automated method for monitoring respiratory behaviors in this species. We propose Mamba-YOLO-SRC, a novel hybrid detection framework that combines Mamba and YOLO architectures to accurately identify four key behaviors: diving (Dive), head-raising (HeadUP), inhalation (Inhale), and exhalation (Exhale). The proposed model achieves a mean average precision (mAP@0.5) of 0.944, with per-class average precision scores of 0.975 for Dive, 0.925 for HeadUP, 0.948 for Exhale, and 0.928 for Inhale. Mamba-YOLO-SRC provides a feasible and referable technical solution for advancing research on the Chinese giant salamander in both captive and natural settings.

## Simple Summary

The Chinese giant salamander is a species of significant conservation and economic value. However, its nocturnal and cave-dwelling habits make it extremely difficult to observe its respiratory behaviors, which can indicate health status. Traditional monitoring methods often miss these subtle behaviors. To address this issue, this study proposes an automated method called Mamba-YOLO-SRC, which accurately identifies four key respiratory behaviors: diving, head-raising, inhalation, and exhalation. Mamba-YOLO-SRC offers a technical reference for advancing research on the Chinese giant salamander in both captive and natural settings.

## 1. Introduction

Breathing, as a core vital sign, sustains animal life by supplying oxygen and concurrently modulating a range of physiological and neurological functions [1]. Rich in diagnostic cues about an individual’s health, it serves not merely as a pivotal metric for monitoring animal well-being [2,3], but also as a lens through which to observe the physiological tactics wildlife deploy to navigate changing environments [4]. Indeed, only by investigating animal respiration can we gain deeper insight into the mechanisms underpinning individual survival adaptations—insights that form a scientific foundation for biodiversity conservation and the design of sustainable management policies.

The Chinese giant salamander (*Andrias davidianus*), belonging to the order Caudata, family Cryptobranchidae, and genus *Andrias* within the class Amphibia [5], is a unique species endemic to China and the largest extant amphibian in the world [6]. Wild populations of the Chinese giant salamander are classified as a Class II nationally protected species in China and listed as Critically Endangered (CR) on the IUCN Red List of Threatened Species [7]. Since the 1950s, their numbers and distribution ranges have sharply declined due to habitat loss and fragmentation caused by human activities. To protect this species, China has established 14 nature reserves since the 1980s, covering a total area exceeding 355,000 hectares [8].

Artificially bred offspring from the second generation onwards are permitted for use as food or nutritional resources [9]. In recent years, with the continuous maturation of artificial breeding technology and expansion of breeding scale, the Chinese giant salamander has gradually become a new breed with market potential. Its unique nutritional and medicinal values show promising prospects for development in food, health supplements, and pharmaceuticals [10,11]. However, increased intensification has led to unavoidable disease outbreaks in artificial breeding, including viral, bacterial, and fungal infections. These pathogens can cause morphological, physiological, and behavioral abnormalities that compromise the health of the Chinese giant salamander. Consequently, disease prevention and control during the breeding process remain the key bottleneck restricting the sustainable development of this industry [12]. Studying their respiratory behavior can help detect and prevent various diseases early, providing a safeguard for artificial breeding. At present, there is a lack of research on the respiratory behavior of *Andrias davidianus*. Early studies mostly focused on the observation of lung respiration during the daytime. For instance, Song et al. [13] described the air-breathing process of this species. Zheng et al. [14] and Chen et al. [15] found that the daily ventilation frequency of *Andrias davidianus* was significantly positively correlated with water temperature under outdoor and indoor breeding conditions, respectively, until Tian et al. [16] systematically monitored the whole-day lung breathing behavior of *Andrias davidianus* and found that individuals in the early reproductive period had two modes of short breathing and long breathing and showed the circadian rhythm characteristics of “active at night and weakened during the day”, which initially filled the gap in the study of the circadian breathing behavior of this species.

The progress of artificial intelligence has propelled computer vision and deep learning to achieve sustained advances, revealing considerable promise in behavioral biology research. In the domain of aquatic animal behavior monitoring, object detection now serves as a cornerstone of automated observation. Leading deep learning detectors—including YOLO and its derivatives, Faster R-CNN, and SSD—have been extensively deployed for localizing, identifying, and analyzing behaviors across diverse aquatic species.

Early research predominantly centered on two-stage detectors, particularly Faster R-CNN and its variants, which laid the foundational framework for subsequent advancements. Li et al. [17] pioneered this trajectory by being the first to apply Faster R-CNN to underwater fish detection, thereby validating the feasibility of deep learning in complex aquatic environments. Building upon this foundation, several studies sought to refine the model’s robustness; notably, Zeng et al. [18] introduced an adversarial occlusion network to mitigate the challenges posed by severe occlusion, while Moniruzzaman et al. [19] successfully adapted the architecture for seagrass detection, demonstrating its versatility in benthic habitat monitoring. Furthermore, the model’s capability was extended beyond mere detection to automation, as evidenced by Gu et al. [20], who employed an enhanced Faster R-CNN to automate the gender identification of juvenile Chinese mitten crabs, effectively addressing the inefficiency of manual sorting. Additionally, Fan et al. [21] explored behavioral analysis by investigating AI-driven behavior recognition in zebrafish through a deep learning-based classification framework.

Consequently, as the demand for real-time processing and deployment on resource-constrained edge devices grew, the research focus gradually shifted toward more efficient single-stage detectors, specifically SSD and its lightweight derivatives. This transition was driven by the need for higher inference speeds without compromising excessive accuracy. Hung et al. [22] effectively applied the SSD-MobileNet architecture to achieve efficient multi-species fish classification, setting a benchmark for lightweight models. Demonstrating the practicality of such models in aquaculture settings, Yu et al. [23] engineered an adaptive dead-fish detection system using SSD-MobileNet, proving its feasibility on hardware with limited computational power. Similarly, Tian et al. [24] devised an improved SSD algorithm specifically optimized to identify fish exhibiting “belly-up” symptoms, a critical indicator of distress in farming environments.

In recent years, YOLO-based architectures have emerged as the dominant paradigm in underwater fish detection, owing to their superior balance between speed and precision. To address the limitations of earlier models, researchers have heavily modified the YOLO framework to suit heterogeneous underwater conditions. Wang et al. [25] integrated an improved YOLOv5 with the SiamRPN++ tracker to enable real-time detection and tracking of abnormal fish behaviors, enhancing temporal analysis. For health monitoring, Wang et al. [26] developed an enhanced YOLOv5 network featuring a context-aware module to facilitate early disease diagnosis in intensive aquaculture. Recognizing the variability of underwater environments, Al et al. [27] introduced YOLO-Fish, a specialized model designed to handle diverse and complex aquatic scenes. More recently, architectural innovations have continued to flourish; Tu et al. [28] presented an upgraded YOLOv8 model based on channel non-degradation mechanisms and spatial coordination attention for general farmed fish monitoring, while Ouis et al. [29] provided a comprehensive evaluation of various YOLO variants under fluctuating turbidity and illumination conditions, offering valuable insights into model robustness.

Yet the efficacy of these detection systems remains fundamentally limited by the harsh realities of underwater imaging. Severe image degradation, which manifests as poor contrast, blurriness, color shifts, and detail loss, substantially undermines detection accuracy and reliability. Consequently, underwater image enhancement has become an indispensable preprocessing stage. Iqbal et al. [30] initially drew upon integrated color models; these efforts were succeeded by Islam et al. [31], who introduced physics-informed rapid enhancement techniques, and more recently by Anwar et al. [32], who proposed end-to-end deep learning strategies. As affirmed by comprehensive reviews from Schettini et al. [33] and Raveendran et al. [34], alleviating underwater image degradation is critical to ensuring dependable object detection and high-fidelity behavioral interpretation in aquatic contexts. Thus, continued innovation in image enhancement remains essential to advancing the precision and robustness of downstream vision tasks in intelligent aquaculture.

While high-quality input images are prerequisite, the ultimate detection performance also hinges on the architectural capacity of the backbone network to efficiently process these enhanced features.

To this end, recent studies confirm that the Mamba architecture, leveraging its linear computational complexity and global receptive field, has significantly overcome the bottlenecks of traditional YOLO models in feature fusion efficiency and long-range dependency modeling. For instance, You et al. [35] proposed Rose-Mamba-YOLO, which effectively addresses occlusion and scale variation challenges in rose detection within complex greenhouse environments, while Xia et al. [36] developed PAB-Mamba-YOLO, utilizing state space models to markedly enhance the accuracy and real-time performance of aggressive behavior detection among weaned piglets. Together, these works highlight the immense potential of integrating advanced state-space models into smart agriculture scenarios, particularly when paired with robust enhancement pipelines.

AIoT and intelligent computing frameworks have demonstrated their potential for real-time prediction and monitoring, and integrating deep learning into heterogeneous computing systems offers a viable pathway for future deployment of animal monitoring technologies [37,38]. Although artificial intelligence-driven target recognition technology has achieved significant results in monitoring various aquatic organisms, its application in the behavioral ecology research of giant salamanders remains largely unexplored. Currently, the observation of respiratory behavior in giant salamanders mainly relies on manual video playback and counting, which is time-consuming, labor-intensive, and susceptible to subjective interference, making it difficult to achieve long-term, high-frequency continuous monitoring, especially for giant salamanders that are highly cryptic and often active at night or inside caves. Consequently, traditional methods have significant limitations in terms of data integrity and objectivity.

Notably, apart from our team’s previously published research on improved YOLOv8-based recognition of juvenile giant salamander behavior [39], there have been no publicly available studies applying YOLO or other mainstream deep learning detection frameworks to the automatic recognition of individual giant salamanders and the precise capture of lung respiration events. In our previous work, we released the first dataset for giant salamander respiratory behavior detection, named CGS-BR (Chinese Giant Salamander Behavior Recognition) [40], which focused primarily on dataset construction and benchmark evaluation of baseline methods. Building upon this dataset, the present study shifts the focus to algorithm development, exploring a target detection model specifically designed for the challenging living environment of giant salamanders, thereby providing a new technical pathway for automated and intelligent behavioral monitoring.

Therefore, this paper innovatively proposes a hybrid model Mamba-YOLO-SRC. Based on the advantages of Mamba and YOLO, SRC module and MVSM module are specially designed, which can effectively detect the lung breathing behavior of *Andrias davidianus*, including diving (Dive), raising head (HeadUP), exhaling (Exhale) and inhaling (Inhale). The main contributions of this study include

- A pioneering deep learning framework for automated respiratory behavior analysis in *Andrias davidianus*. This study establishes the first automated observation methodology for identifying lung breathing patterns in giant salamanders. The proposed approach not only advances the application of information technology in amphibian behavioral ecology but also provides a technical reference for intelligent behavioral analysis in other amphibious and aquatic species.
- A novel dual-branch Super-Resolution Fusion Component (SRC) for low-contrast scene enhancement. By integrating the SRCNN super-branch with a Simple Stem trunk, it restores high-frequency image details early in feature extraction, effectively enhancing target perception and significantly reducing miss detection rates under low-light conditions or when the target blends into the background.
- A Multi-scale Visual Spatial Module (MVSM) is introduced to strengthen behavioral feature extraction. Specifically, the MVSM leverages a combination of multi-scale convolutions and a spatial attention mechanism to capture richer features related to postural changes, thereby improving the model’s sensitivity to subtle respiratory motions.

## 2. Materials

### 2.1. Data Sources

**Figure 1:**
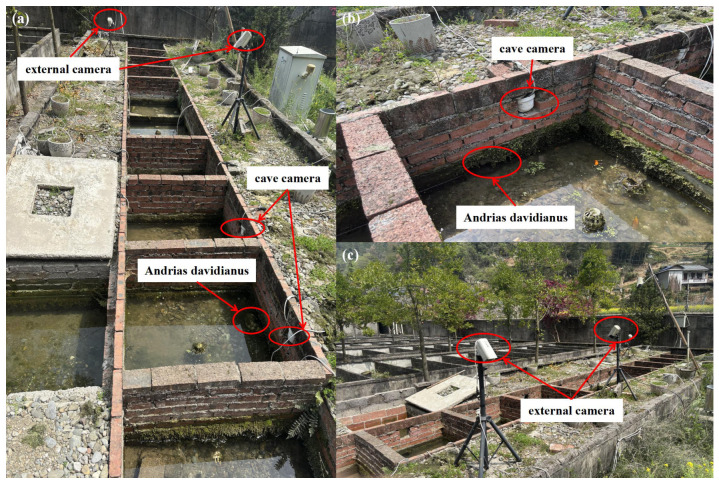
Simulated natural *Andrias davidianus* feeding pond. (**a**) External overview; (**b**) Cave camera view; (**c**) External side view.

The CGS-BR dataset was constructed using experimental data collected from the ecological breeding base of Zhuyuan *Andrias davidianus* Biotechnology Co., Ltd. in Kongshu Township, Sangzhi County, Zhangjiajie City, Hunan Province, China (29°28′ N, 110°22′ E, 471 m above sea level). The facility features artificial streams (approximately 22 m long, 1.10 m wide, and 0.40 m deep) and caves. Each cave has an area of about $1.44\;m^{2}$, a height of about 0.25 m, a width of about 0.30 m, and a depth of about 0.20 m, with the bottom layer covered by sand and cobbles. One female and one male giant salamander, both wild F2-generation individuals, are housed in each cave. A total of 12 individuals (6 males and 6 females) were included in this dataset. Their body lengths range from 1.12 m to 1.26 m, body weights from 9.80 kg to 12.20 kg, and ages from 9 to 10 years. Infrared cameras (Hikvision DS-2CD3T35D-I5, Hangzhou Hikvision Digital Technology Co., Ltd., Hangzhou, China) were installed at the observation opening and above the cave of the imitation ecological breeding pool, as shown in Figure 1, to record the breathing behavior and other activities of the giant salamanders both inside and outside the caves. Data were collected from December 2022 to November 2023, covering a full annual cycle, including seasonal variations, different physiological states, and diverse environmental lighting conditions. Continuous 24 h daily recording of *Andrias davidianus* behavior was performed. Video recordings were obtained in HEVC format with a resolution of 2560×1040 pixels and a frame rate of 50 fps.

### 2.2. Dataset Construction

**Figure 2:**
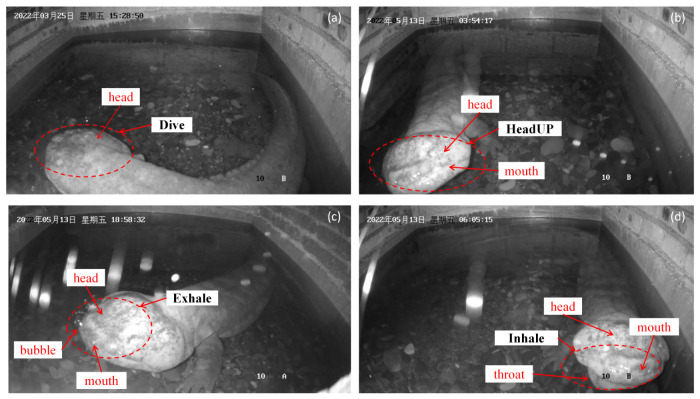
Example of dataset for lung breathing behavior of *Andrias davidianus*. (**a**) Dive; (**b**) HeadUP; (**c**) Exhale; (**d**) Inhale.

The definition of lung breathing behavior of giant salamanders in this study is as follows [13,14]: “The giant salamander raises its head above the water to inhale, then lowers its head and dives into the water to exhale, which is considered a complete lung breathing behavior.” Accordingly, we divide this behavior into four actions, head raising, inhaling, diving, and exhaling, as illustrated in Figure 2.

**Figure 3:**
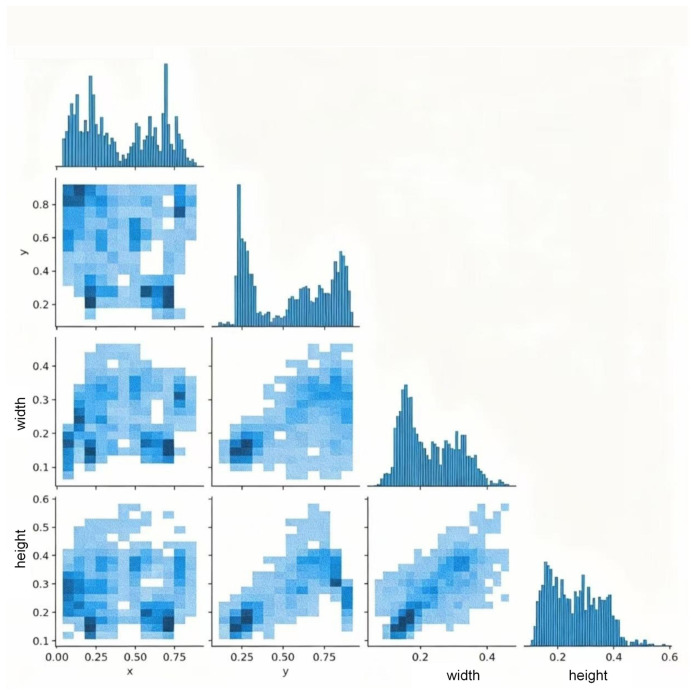
Distribution of normalized bounding box parameters in the *Andrias davidianus* dataset. The histograms show the distributions of normalized x-coordinate, y-coordinate, width, and height.

We extracted 1732 images from 215 video clips and divided them into training, test, and validation sets (see Table 1). We performed a stratified split on the CGS-BR dataset as follows. First, frames were sampled at regular intervals from the continuous video recordings. Second, based on the distribution of the four respiratory behavior categories, we ensured that the training, validation, and test sets each maintained a class distribution similar to that of the original dataset. Meanwhile, we adopted a video-level partitioning principle, taking each video clip recording a complete respiratory behavior as the basic unit, and assigning all image frames extracted from the same clip collectively to one of the subsets. This prevents highly similar consecutive frames from appearing across different subsets, thereby avoiding data leakage and falsely inflated model performance. The lung breathing behavior regions in the images were annotated by giant salamander experts using the open-source tool Label Studio (https://labelstud.io/, accessed on 1 March 2026). All annotations were reviewed and verified by a senior expert from the same laboratory to ensure quality. Figure 3 shows the distribution of annotated bounding box locations and sizes, which facilitates understanding of dataset characteristics and model performance evaluation.

## 3. Methodology

### 3.1. Overall Architecture

**Figure 4:**
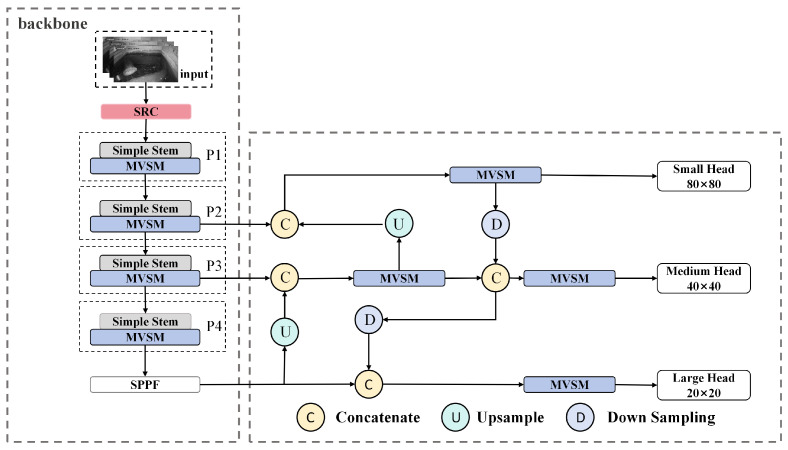
The Mamba-YOLO-SRC Model.

The Mamba-YOLO-SRC hybrid model proposed in this study is mainly composed of three core components: backbone network, neck network and detection head. The model systematically integrates Mamba state space model, visual cue fusion mechanism and multi-scale visual space modeling strategy. In the backbone network part, this research model discards the traditional structure and innovatively introduces the Multi-scale Visual Spatial Module (MVSM) to replace the C2f module in the original YOLOv8n architecture. MVSM can enhance the ability to capture subtle motion features by building a cross-level spatial attention mechanism and gradient perception convolution path. In addition, in order to deal with the problems of low resolution of the input image and feature degradation caused by texture blur, this study designs a Super-Resolution Fusion Component (SRC), which is integrated into the front end of the backbone network. SRC is mainly composed of two parts. Firstly, SRCNN sub network performs super-resolution reconstruction on the input image to recover the lost high-frequency details. Then, the simple stem structure is responsible for channel compression and spatial dimensionality reduction to effectively reduce the subsequent computer overhead. The neck network adopts PAFPN structure, which is responsible for effective feature aggregation and transmission of multi-level feature maps {C1, C2, C3, C4, C5} from the backbone network, and strengthens the reverse injection of semantics into low-level features. The detection head uses decoupling detection head to perform the boundary box and classification tasks respectively. Each task has an independent parameter path, avoiding the mutual gradient interference between multiple tasks, and achieving precise division of labor in positioning and classification tasks, as shown in Figure 4.

### 3.2. Visual State-Space Model

Mamba is a sequence modeling architecture based on Structured State Space Models (SSMs), designed to overcome the computational bottlenecks of traditional recurrent neural networks and attention mechanisms in long-sequence processing. Compared to standard SSMs, Mamba introduces a selective state update mechanism, enabling it to dynamically adjust the state evolution path based on input content. This allows Mamba to focus on critical information, enhancing feature representation capability while maintaining linear time complexity [41]. In continuous time, an SSM maps a one-dimensional input sequence $x\left(t\right)\in R^{C}$ to a hidden state sequence $h\left(t\right)\in R^{N}$ through dynamic propagation, described by a set of linear ordinary differential equations (ODEs):

(1) $$\begin{array}{cc} h^{\prime }\left(t\right) & =Ah(t)+Bx(t) \end{array}$$

(2) $$\begin{array}{cc} y(t) & =Ch(t)+Dx(t) \end{array}$$

Here, $A\in R^{N\times N}$ is the state transition matrix, governing the internal dynamics of the hidden state; $B\in R^{N\times C}$ and $C\in R^{C\times N}$ are the input projection matrix and output projection matrix, respectively; and $D\in R^{C\times C}$ is the feed-through matrix, preserving the direct response from the input [42]. This system essentially constitutes a linear time-invariant (LTI) dynamic system, capable of performing global context modeling over the input sequence. In deep learning applications, since input data is presented as discrete sequences, the zeroth-order hold assumption is typically adopted—assuming the input signal remains constant within each sampling interval [kΔ,(k+1)Δ]—enabling an equivalent conversion from the continuous-time dynamics to a discrete-time update rule [43].

These fixed discretization rules serve as the foundation for SSM applications, enabling Mamba to be seamlessly integrated into deep learning frameworks.

### 3.3. Super-Resolution Fusion Component

**Figure 5:**
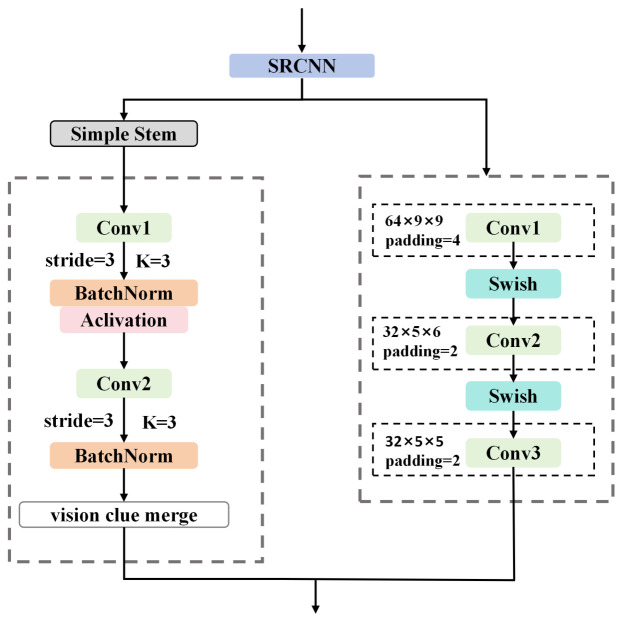
SRC Module: From SRCNN splitting into Simple Stem and SRCNN branches, then fusing.

In complex scenes, the Chinese giant salamander exhibits strong camouflage due to its coloration being highly similar to the surrounding environment. The target often suffers from low contrast and sparse pixel information, leading to weak feature responses and easy loss in deep network layers. To mitigate this issue, we propose a Super-Resolution Fusion Component (SRC), designed to restore high-frequency details lost at the image input stage and enhance the detectability of small targets in feature space through an adaptive fusion mechanism. The SRC consists of two core components: a super-resolution reconstruction subnetwork (SRCNN) [44] and a lightweight dimensionality-reduction structure (Simple Stem). By fusing multi-level features from the super-resolution branch and the main backbone network, SRC strengthens the representational capacity of small targets in feature space, as illustrated in Figure 5.

Modern visual transformers (ViTs) typically use patch embedding as the initial module to extract features by dividing the input image into non-overlapping image blocks. This process is typically accomplished through convolution operations with a kernel size of 4 and a stride of 4. However, recent studies have pointed out that such designs may limit the optimization potential of the model, thereby affecting overall performance [45]. To achieve a better balance between computational efficiency and model capability, this paper proposes a simplified dry layer structure: abandoning the traditional non-overlapping block partitioning method and instead using two consecutive 3 × 3 convolutional layers, each layer using a stride of 2 convolution kernels, to achieve smoother spatial downsampling and better feature preservation.

Moreover, Liu et al. [46] observed that both Convolutional Neural Networks (CNNs) and Vision Tree Structures (ViVTS) commonly employ convolutional layers for downsampling, which can interfere with the precise information processing mechanism of SS2D across different stages of feature flow. Concurrently, Wang et al. [47] recently proposed splitting 2D feature maps to preserve more visual cues beneficial for small-object detection, and introduced a Vision Clue Merge module that improves model performance. Notably, this idea aligns closely with recent advances in neurodynamics focusing on preserving critical information under noisy conditions. For instance, in Xiao et al. [48] and Liao et al. [49], the authors introduced predefined-time convergence mechanisms and harmonic-noise suppression structures, respectively, which effectively guarantee the integrity of weak yet essential signals in dynamic systems—precisely analogous to the preservation of high-frequency details in low-contrast small-object detection. Inspired by such robust neural modeling paradigms, our design of the lightweight dimensionality-reduction structure (Simple Stem) not only incorporates the Vision Clue Merge module from Wang et al. [47], which preserves SS2D-selected visual clues by replacing traditional stride convolutions with norm removal, channel splitting, feature appending, and 4× compressed pointwise convolution, but also further draws upon the ZNN framework’s principle of “prioritizing dominant feature components under interference” to effectively retain high-frequency details crucial in low-contrast scenarios.

(3) $$F_{\mathrm{merge}}=\alpha \;\cdot \;F_{\mathrm{backbone}}+\beta \;\cdot \;U\left(F_{s}\right),$$

where α and β denote learnable weights initialized to 1 and optimized during training to adaptively balance the influence of distinct pathways. $F_{\mathrm{backbone}}$ represents the features extracted by the left branch for channel compression and spatial downsampling. $F_{s}$ denotes the output of the upsampling layer in the right branch, which provides high-frequency details. $U(F_{s})$ signifies a spatial or channel-wise transformation applied to $F_{s}$ to align its dimensionality and scale with $F_{\mathrm{backbone}}$. The enhanced feature representation $F_{\mathrm{merge}}$ is generated via this weighted combination.

(4) $$F_{\mathrm{SRC}}=\sigma \left(F_{\mathrm{merge}}+F_{\mathrm{srcnn}}\right),$$

where $F_{\mathrm{srcnn}}$ refers to the final output features of the SRCNN branch after subsequent convolutional processing, which is distinct from $F_{s}$ used in Equation (3). σ stands for the Swish activation function, introducing nonlinearity to enhance the model’s representational capacity.

By adopting the above strategies, our method not only reduces data dimensionality but also preserves critical visual information, thereby improving detection accuracy in scenarios where the target exhibits low contrast against the background.

### 3.4. Multi-Scale Visual Spatial Module

Based on the selective scanning mechanism, the Vision State Space Module (VSSM) can effectively focus on key regions of the input data and extract task-relevant features. However, when used as the basic building block of a backbone network, VSSM may suffer from limited local receptive fields and insufficient feature diversity in deep layers. To address these limitations, we propose an improved state space module—Multi-scale Visual Spatial Module (MVSM)—designed to enhance feature representation capability and model learning efficiency through structural optimization.

**Figure 6:**
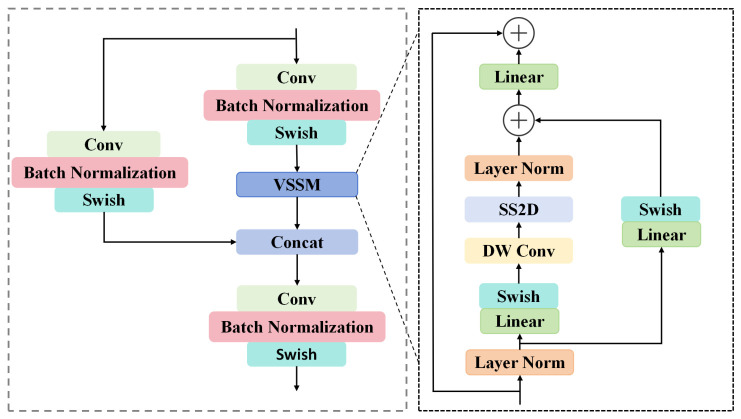
MVSM Module: dual branches for local detail preservation (**left**) and global context modeling (**right**), with cross-branch fusion and residual connection.

Figure 6 illustrates local detail preservation and long-range dependency modeling inspired by Xia et al. [36]. The input features are first normalized through layer normalization and then evenly divided into two complementary feature subsets, which are respectively sent to the left and right functionally heterogeneous processing paths. In the left pathway, a lightweight standard convolution structure is adopted, which includes a convolutional layer, batch normalization, and Swish activation function, focusing on quickly capturing local spatial patterns and maintaining the detail integrity of the feature map. On the right side, an enhanced state space processing chain is constructed, which includes linear transformations, depthwise separable convolutions, and nonlinear activation functions. Finally, a two-dimensional selective module (SS2D) is integrated to achieve efficient modeling of the global spatial context. The output feature maps of the two channels are concatenated in the channel dimension, and cross-channel information fusion and dimension alignment are achieved through a subsequent standard convolution layer. This design not only promotes the deep fusion of local and global features, but also enhances the model’s perception ability of multi-scale targets. In addition, MVSM introduces a residual connection structure to transfer input features to the output through shortcut paths, thereby alleviating the problem of gradient vanishing and improving training stability. The entire module significantly enhances the richness and robustness of feature representation while maintaining low computational overhead.

The output feature maps from the two branches are concatenated along the channel dimension and subsequently processed by a standard convolutional layer to enable cross-branch information fusion and dimensional alignment. This process can be formulated as

(5) $$F_{\mathrm{fuse}}={\mathrm{Conv}}_{\mathrm{fuse}}\left(\left[F_{\mathrm{left}},\;F_{\mathrm{right}}\right]\right),$$

where $F_{\mathrm{left}}$ and $F_{\mathrm{right}}$ denote the output features from the left local branch and the right global modeling branch, respectively. ${\mathrm{Conv}}_{\mathrm{fuse}}$ is a convolutional layer used to fuse cross-branch features and align the channel dimension.Furthermore, MVSM incorporates a residual connection that bypasses the main path via a shortcut, allowing the input feature to be directly transmitted to the output. This design alleviates the gradient vanishing problem and enhances training stability. The final output of MVSM is defined as

(6) $$Y=F_{\mathrm{fuse}}+X.$$

where X denotes the input feature map to the MVSM module, and Y represents its enhanced output feature map.

This design facilitates the effective integration of local and global features, thereby improving the model’s ability to perceive multi-scale objects. The module enhances feature representation, while maintaining reasonable computational overhead.

## 4. Implementation Details and Results Analysis

### 4.1. Experimental Settings

The Mamba-YOLO-SRC model proposed in this study was trained on a cloud server configured with an NVIDIA RTX 4090D graphics card (24 GB of VRAM) and 80 GB of RAM. The software environment includes Ubuntu 20.04 LTS system, CUDA 12.1 driver, Python 3.12 programming language, and PyTorch 2.3.0 deep learning framework. The specific configuration of the experimental environment is detailed in Table 2. The input image is uniformly cropped to a size of 640×640, with a batch size of 32 and a training period of 120 epochs. The model was optimized using the SGD optimizer with an initial learning rate of 0.01, momentum of 0.937, and weight decay of 0.0005. A cosine annealing learning rate scheduler was adopted. Data augmentation included random flipping, mosaic, and scaling. A fixed random seed of 42 was used for reproducibility.

### 4.2. Evaluation Metrics

To objectively evaluate the performance of the proposed method, this study adopts a comprehensive set of evaluation metrics, number of model parameters (Params), floating-point operations in gigabytes (GFLOPs), average precision (AP), and mean average precision (mAP), which are used to assess the model’s accuracy. These metrics collectively reflect the model’s scale, computational complexity, and detection efficiency.

TP denotes the number of target frames correctly predicted as positive instances, FP represents the number of target frames incorrectly classified as positive (false alarms), and FN denotes the number of positive instances that are misclassified as negative (missed detections).

Precision is the ratio of true positive detections to all positive predictions made by the model, defined as

(7) $$\mathrm{Precision}=\frac{TP}{TP+FP}$$

Recall is the ratio of the number of targets correctly predicted as positive instances to the total number of actual positive instances, defined as

(8) $$\mathrm{Recall}=\frac{TP}{TP+FN}$$

AP is the area under the precision–recall curve, representing the average precision of the model across different recall levels, defined as

(9) $$\mathrm{AP}=\int_{0}^{1}\mathrm{Precision}\;d\left(\mathrm{Recall}\right)$$

mAP is a comprehensive metric used to evaluate the performance of object detection models across multiple categories. It computes the average precision (AP) for each class and then takes the mean of these AP values to provide an overall measure of model performance:

(10) $$\mathrm{mAP}=\frac{\sum_{i=1}^{N}\mathrm{AP}\left(i\right)}{N}$$

where *N* denotes the number of classes in the dataset, and in this study, N=4. A higher mAP value indicates better model performance.

### 4.3. Experimental Results

**Figure 7:**
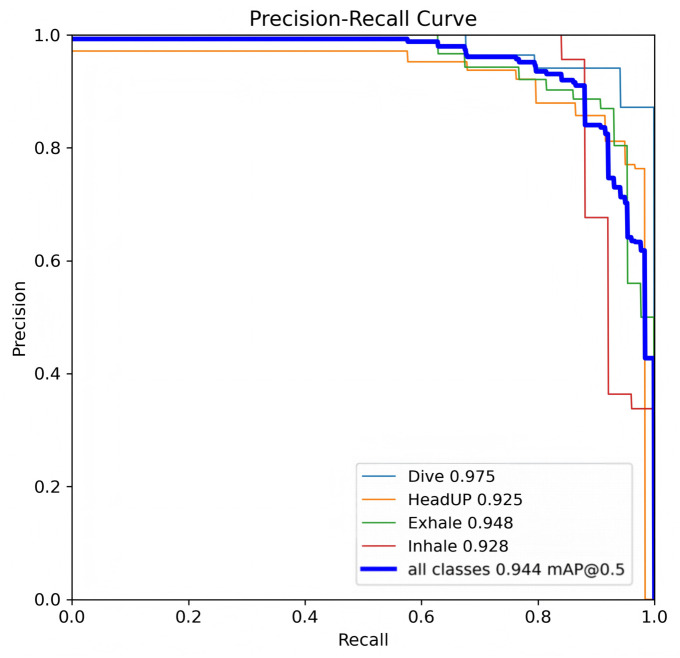
Precision–recall (PR) curve: evaluating the model’s performance.

Our model achieves an accuracy characterized by the average precision (AP) values for the four behavioral classes of the Chinese giant salamander (Dive, HeadUP, Exhale, and Inhale), which are 0.975, 0.925, 0.948, and 0.928, respectively. For a comprehensive evaluation of these four respiratory behaviors, the model attains a mean average precision (mAP) of 0.944. Figure 7 presents the precision–recall (P-R) curves of the proposed model on the test set. As can be seen, the Mamba-YOLO-SRC model exhibits excellent training performance.

**Figure 8:**
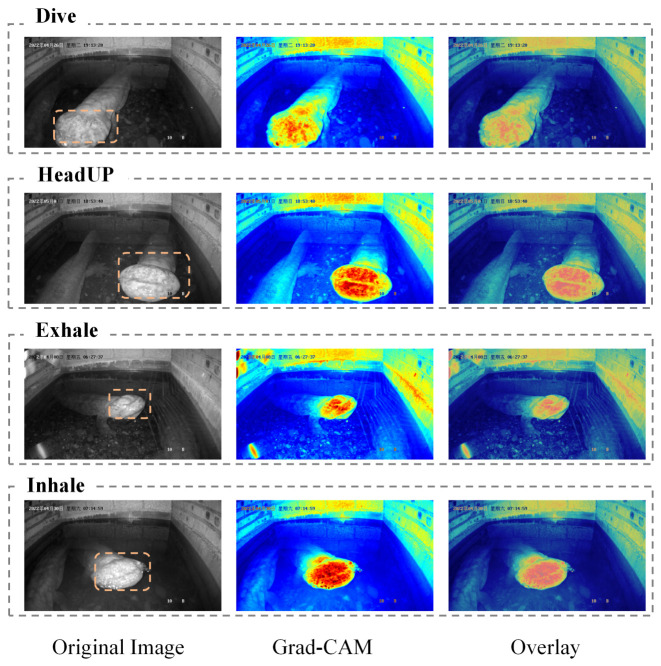
Grad-CAM heatmaps: highlighting the model’s focus on respiratory regions of *Andrias davidianus*. Warmer colors indicate higher attention, while cooler colors indicate lower attention.

To further understand the decision-making mechanism of the model in recognizing the respiratory behaviors of *Andrias davidianus*, we employed Grad-CAM [50] to visualize the feature responses of the Mamba-YOLO-SRC model. As shown in Figure 8, the heatmaps reveal that the model’s attention is predominantly focused on the head and snout regions of *Andrias davidianus*, indicating that the model effectively focuses on the key body parts relevant to respiratory behavior.

### 4.4. Ablation Experiments

To verify the effectiveness of the optimized components in the Mamba-YOLO-SRC model, we conduct an ablation study on the same test set. The original YOLOv8n model is adopted as the baseline, and the MVSM and SRC modules are incrementally integrated into the baseline for performance evaluation. The detailed comparison results are presented in Table 3.

1. By comparing Group 1 and Group 2, we observe that the proposed SRC module improves detection accuracy, with mAP@0.5 rising from 0.923 to 0.931. The parameter count increases slightly from 3.12 M to 3.31 M.
2. Comparing Group 1 and Group 3, we find that integrating the MVSM module alone boosts mAP@0.5 to 0.937, with GFLOPs increasing from 8.2 to 13.6 and parameters from 3.12 M to 5.82 M.
3. Finally, by incorporating both SRC and MVSM modules into the baseline, we construct the complete Mamba-YOLO-SRC model. Experimental results show that mAP@0.5 reaches 0.944, a 2.1 percentage point improvement over the original YOLOv8n (0.923), demonstrating the effectiveness of the proposed method. Notably, due to the introduction of additional modules, the FPS decreases from 112.6 to 47.2, and GFLOPs increase to 28.1. The 2.1 percentage point improvement in mAP@0.5 is practically meaningful, particularly for challenging tasks such as giant salamander respiratory monitoring. While this gain comes with increased computational cost, the trade-off between accuracy and efficiency depends on the deployment scenario. The proposed model is preferable when detection accuracy is prioritized, whereas other lightweight models remain more suitable choices when real-time throughput is critical.

### 4.5. Comparative Experiment

To evaluate the performance of the proposed model, we conduct a comprehensive comparison with a variety of representative object detection models. The experiments encompass mainstream architectures based on both Convolutional Neural Networks (CNNs) and Transformers, aiming to comprehensively assess the performance of the proposed method across different technical paradigms and model complexities.

Specifically, we select YOLOv5n [51], YOLOv7-tiny [52], YOLOv8n [53], and YOLOv10n [54] as representatives of lightweight CNN single-stage detectors. These models are widely used in real-time behavior recognition tasks due to their efficient convolutional operations. Meanwhile, to explore the potential of pure Transformer or hybrid architectures, we introduce the classic CNN two-stage detector represented by Faster R-CNN, along with advanced detectors such as Ddod [55], VFNet [56], and RT-DETR-R18 [57] (where RT-DETR represents a Transformer-based end-to-end detection paradigm). This comparative scheme not only covers the trade-off between accuracy and speed but also reflects the evolution from traditional convolutional feature extraction to self-attention mechanism-based localization capabilities, thereby facilitating a comprehensive evaluation of the proposed method.

All baseline models were trained with their official default configurations, and no architecture-specific hyperparameter tuning was performed to ensure a fair comparison of out-of-the-box performance. Each model was then trained and evaluated on the same A. davidianus respiratory behavior dataset with identical input resolution (640×640). The number of parameters (Params) and computational costs (GFLOPs) are measured on the same hardware platform (NVIDIA RTX 4090D) to ensure fair and reproducible comparisons.

As shown in Table 4, in terms of average detection precision, Mamba-YOLO-SRC achieves a mAP@0.5 of 0.944, surpassing Faster R-CNN (0.713), Ddod (0.915), VFNet (0.909), RT-DETR-R18 (0.916), YOLOv5n (0.913), YOLOv8n (0.923), YOLOv10n (0.920), and YOLOv7-tiny (0.880), indicating its stronger capability in high-confidence object recognition. For the Dive and Exhale behaviors, the proposed model achieves AP scores of 0.975 and 0.948, respectively, outperforming the other baseline models. On HeadUP and Inhale, its performance is similar to that of the other baseline models.

**Figure 9:**
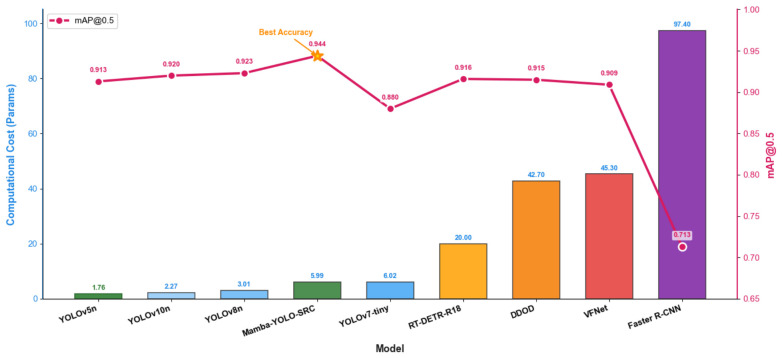
Comparison of computational cost (GFLOPs) and detection accuracy (mAP@0.5) among different models.

As shown in Figure 9, our Mamba-YOLO-SRC achieves the highest mAP@0.5 (0.944) with 5.99 GFLOPs. In comparison, YOLOv5n has lower computational cost (1.76 GFLOPs) but also lower accuracy (0.913). Overall, Mamba-YOLO-SRC achieves a relatively balanced trade-off between computational efficiency and detection accuracy.

**Figure 10:**
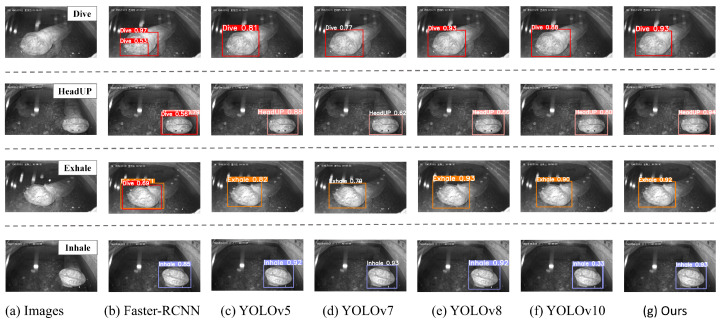
Comparison of detection results. All bounding boxes use a consistent color scheme for fair comparison.

As shown in Figure 10, we present a visual comparison of detection results across different models for the four respiratory behaviors of the Chinese giant salamander: Dive, HeadUP, Exhale, and Inhale. Since the proposed Mamba-YOLO-SRC is built upon the YOLO architecture, we primarily select representative YOLO-series models (YOLOv5n, YOLOv7-tiny, YOLOv8n, YOLOv10n) for visual comparison to more intuitively verify the improvement, while also including Faster R-CNN as a reference for two-stage detectors.

Faster R-CNN suffers from missed detections and false positives under complex backgrounds and low-contrast conditions, particularly showing low confidence in subtle actions such as Exhale and HeadUP. In contrast, YOLOv5n, YOLOv7-tiny, and YOLOv10n do not exhibit obvious false detections, yet still show relatively low confidence in recognizing certain respiratory behaviors. YOLOv8n demonstrates high overall confidence across all behavior categories, but remains inadequate in detecting the HeadUP behavior—a critical action for pulmonary respiration. By comparison, the proposed Mamba-YOLO-SRC model achieves high-confidence and accurate detection across all behavior types.

In summary, compared with Faster R-CNN, Ddod, VFNet, RT-DETR-R18, YOLOv5n, YOLOv7-tiny, YOLOv8n, and YOLOv10n, Mamba-YOLO-SRC achieves the highest mAP@0.5 (0.944) with moderate computational cost (5.99 GFLOPs). It demonstrates solid performance across all four behavior categories: Dive (0.975), HeadUP (0.925), Exhale (0.948), and Inhale (0.928). Experimental results on the CGS-BR dataset validate the feasibility of Mamba-YOLO-SRC for respiratory behavior analysis in captive giant salamanders.

## 5. Discussion

### 5.1. Research Value

The primary value of this study is to explore the feasibility of applying deep learning-based object detection to automatic recognition of respiratory behaviors in the giant salamander (*Andrias davidianus*). To this end, we propose Mamba-YOLO-SRC, which improves the efficiency of long-term behavioral observation in captive environments and overcomes the limitations of manual recording in time coverage and data accuracy. Experimental results show that our method captures respiratory changes under specific environmental conditions, providing technical support for optimizing captive management of *Andrias davidianus*. In practice, this automated approach can be deployed by processing video streams from standard surveillance cameras installed in captive facilities. The model analyzes each video frame in real time to detect and classify respiratory behaviors including HeadUP, Inhale, Dive, and Exhale. Detected events are logged with timestamps, and when abnormal patterns are identified, timely feedback can be provided. This enables continuous, objective monitoring of respiratory behavior, facilitating early detection of abnormalities and reducing reliance on manual observation.

### 5.2. Limitations and Prospects

Since the current dataset was collected from a captive environment and includes a limited number of individuals (12), the model trained on this data may learn features that reflect a combination of respiratory behavior and individual- or facility-specific visual patterns. Consequently, its generalization to wild or visually complex scenes remains uncertain. Regarding the model, although Mamba-YOLO-SRC achieves competitive detection accuracy, further analysis using Grad-CAM reveals weak responses in certain background regions within the heatmaps, indicating that the model does not fully focus on the discriminative respiratory regions and still exhibits some dependence on environmental context, which may be attributed to the relatively uniform background of the captive environment. To address this limitation, subsequent work will focus on improving generalization to wild scenarios. Specifically, we will perform a quantitative evaluation of the alignment between Grad-CAM heatmaps and annotated respiratory regions (e.g., calculating the IoU between heatmap activation areas and bounding boxes). Meanwhile, to tackle the class imbalance in the current dataset, we plan to collect more video samples from multiple wild populations of giant salamanders across different geographic regions, thereby expanding the dataset with more diverse backgrounds, individual variations, and more balanced class distributions. We will also explore class-balanced loss functions or targeted data augmentation methods to improve recognition performance on minority classes. Furthermore, we will make further improvements to guide the model to focus on key respiratory regions (e.g., mouth and throat) while reducing background interference, thereby enhancing detection robustness when transferring to complex, low-light wild environments.

## 6. Conclusions

This study presents the first application of artificial intelligence technology to automated respiratory behavior detection in the Chinese giant salamander. The proposed Mamba-YOLO-SRC achieves a mAP@0.5 of 0.944 with moderate computational cost on the CGS-BR dataset, demonstrating solid detection accuracy. Although current limitations remain in dataset scale and scene generalization, future work will focus on expanding monitoring coverage across different habitats and integrating multi-population data to further enhance model robustness. We hope that this technology can provide effective technical support for the captive management and behavioral monitoring of the Chinese giant salamander in the future.

## Figures and Tables

**Table 1:** Definition of lung breathing behavior of *Andrias davidianus* and dataset statistics.

Behavior	Definition	Train	Val	Test	Total
HeadUP	*Andrias davidianus* raises its head above water, mouth closed, no throat expansion	467	88	85	640
Inhale	*Andrias davidianus* opens mouth and pharynx, inhales air, throat visibly expands	203	38	37	278
Dive	*Andrias davidianus* submerges its head under water, nostrils submerged, no breathing action in this frame	267	50	48	365
Exhale	*Andrias davidianus* exhales air from lungs underwater, bubbles visible, bubbles released from mouth or nostrils	335	64	50	449

**Table 2:** Training environment and hardware parameter platform.

Category	Configuration
CPU	18 vCPU AMD EPYC 9754 128-Core Processor
GPU	RTX 4090D (24G)
System environment	Ubuntu 20.04
Framework	PyTorch 2.3.0
Programming language	Python 3.12

**Table 3:** Ablation study results (bold text indicates newly added or replaced modules in each configuration).

Method	Neck	Backbone	mAP@0.5	GFLOPs	Params ($10^{6}$)	FPS
YOLOv8n		C2f	0.923	8.2	3.12	112.6
	**+SRC**	C2f	0.931	22.7	3.31	58.4
		**MVSM**	0.937	13.6	5.82	93.8
	**+SRC**	**MVSM**	0.944	28.1	5.99	47.2

**Table 4:** Comparative Evaluation of Model Performance on CGS-BR Dataset.

Method	Dive	HeadUP	Exhale	Inhale	mAP@0.5:0.95	mAP@0.5	Params	GFLOPs	FPS
Faster R-CNN	0.647	0.751	0.724	0.733	0.643	0.713	97.4	41.36	7.5
DDOD	0.902	0.926	0.912	0.920	0.688	0.915	42.7	32.20	35.2
VFNet	0.908	0.912	0.904	0.913	0.695	0.909	45.3	32.72	32.8
RT-DETR-R18	0.912	0.918	0.914	0.920	0.721	0.916	20.0	60.91	45.5
YOLOv5n	0.892	0.928	0.916	0.921	0.690	0.913	1.76	4.1	124.5
YOLOv7-tiny	0.862	0.918	0.882	0.858	0.642	0.880	6.02	13.2	98.3
YOLOv8n	0.916	0.886	0.938	0.952	0.710	0.923	3.01	8.2	112.6
YOLOv10n	0.908	0.932	0.916	0.924	0.698	0.920	2.27	8.4	108.4
Ours	0.975	0.925	0.948	0.928	0.718	0.944	5.99	28.1	47.2

## Data Availability

The data are not publicly available due to the privacy policy of the organization.
